# Examining Trajectories of Change on the Dynamic Risk Assessment for Offender Re-Entry (DRAOR)

**DOI:** 10.1177/0306624X241240701

**Published:** 2024-04-05

**Authors:** Danielle J. Rieger, Bronwen Perley-Robertson, Ralph C. Serin

**Affiliations:** 1 Carleton University, Ottawa, ON, Canada

**Keywords:** risk assessment, dynamic risk, change, DRAOR, multilevel mixed model, measurement invariance, predictive validity

## Abstract

Dynamic risk scales have largely been evaluated using singular assessment scores, including those obtained at the start of supervision. While this approach includes assessment of dynamic factors, it ignores changes with reassessment, failing to examine whether an instrument is truly dynamic in nature. This is problematic, as proximal risk assessments have consistently outperformed baseline assessments in the prediction of recidivism. In the current study, we examined the dynamic properties of the Dynamic Risk Assessment for Offender Reentry (DRAOR) in 4,736 adults on community supervision in Iowa, United States (*N* = 33,965 assessments). As expected, while clients demonstrated statistically significant changes on the DRAOR domains over time, changes were small in magnitude. We also examined the predictive validity of baseline and proximal DRAOR total and domain scores on criminal recidivism and revocation in a larger sample of 11,421 adults in the same jurisdiction. While DRAOR baseline scores did predict both outcomes, prediction did not improve with proximal scores. This conflicted with expected findings from previous research on the DRAOR in New Zealand. The results of both of these research questions indicate there was an overall lack of change reflected in this sample. Potential issues regarding implementation fidelity are discussed. Additional research is needed to examine the dynamic properties of the DRAOR in Iowa given the importance of reassessment data in community corrections.

The assessment of recidivism risk is vital to the effective management of justice-involved persons and therefore has important public safety implications. To accurately assess risk, correctional agencies must identify both who is likely to reoffend and when they are likely to do so. Historically, this has involved measuring the presence and timing of variables linked to recidivism. These variables—known as risk factors—have been organized into a three-tiered system based on their volatility and proximity to criminal behavior (Lloyd, 2015). First are static risk factors, which are fixed and the most distal to recidivism (e.g., criminal history; Bonta, 1996; Zamble & Quinsey, 1997). These variables can be used to determine someone’s baseline level of risk and therefore represent risk status (Douglas & Skeem, 2005). Next are dynamic risk factors, which are changeable and more proximal to recidivism (Bonta, 1996; Kraemer et al., 1997; Zamble & Quinsey, 1997). Increases or decreases in these variables lead to corresponding changes in criminal behavior, meaning they serve as both risk indices and intervention targets.

The intervention strategy needed to address dynamic risk factors depends on their temporal relationships with recidivism. Therefore, dynamic risk factors can be further classified as stable or acute. Stable dynamic risk factors are relatively enduring, with changes typically occurring over months or years (e.g., impulse control, attitudes toward authority; Hanson & Harris, 2000). These factors are best conceptualized as clinical targets within rehabilitation programs that can be addressed over the long term (Lloyd, 2015). Conversely, acute dynamic risk factors change more rapidly, usually over days, hours, or even minutes (e.g., anger/hostility, negative mood; Hanson & Harris, 2000). These factors represent circumstances, behaviors, and characteristics that signal imminent offending; thus, they are highly proximal to criminal behavior and represent risk state (Lloyd, 2015). Indeed, acute risk factors have been shown to predict imminent recidivism more strongly than stable risk factors, suggesting they require more immediate attention when present (Lloyd et al., 2020).

Both static and dynamic risk factors are useful for predicting recidivism but focusing rehabilitation efforts solely on risk may discount the positive aspects of one’s life that decrease their likelihood of reoffending. Risk assessment and management practices have expanded in recent decades to also incorporate strengths to allow for a more positive approach to rehabilitation. Strengths represent internal or external assets that are both changeable (Ullrich & Coid, 2011) and that mitigate criminal behavior (e.g., prosocial identity/support; Cox et al., 2018; Haines et al., 2018; Jones et al., 2015; Lodewijks et al., 2010; Lowder et al., 2017; Persson et al., 2017; Viljoen et al., 2016; Yoon et al., 2018).

Despite the widespread use of dynamic risk factors in corrections, there is limited evidence showing that they are truly dynamic in nature (i.e., that they change, and that these changes predict recidivism; Lloyd et al., 2020). There are also few studies on the dynamic nature of strengths. Many studies use a pre-post follow-up design to examine changes in risk and strengths (e.g., Beggs & Grace, 2011; de Vries Robbé et al., 2011; Hogan & Olver, 2019; Lovatt et al., 2022; Olver et al., 2020; Rojas & Olver, 2022), but with only two assessments, it is difficult to determine whether change is merely the result of measurement error (Cronbach & Furby, 1970; Babchishin & Hanson, 2020). Fortunately, there is a burgeoning body of research aimed at filling this gap in the literature. One relatively early study examined change on the Offender Assessment System (OASys) in a large community-based sample assessed every 3 months for a maximum of 4.5 years (Howard & Dixon, 2013). Reassessments were found to predict nonsexual violent recidivism in the subsequent 3-month period more strongly than baseline assessments conducted at release from prison.

More recently, Babchishin and Hanson (2020) examined change on the ACUTE-2007 in a sample of men convicted of sexually motivated offences. During the 19-month assessment period, wherein 2 to 69 assessments were obtained per person, the sample demonstrated significant changes in acute risk. The latest score or a rolling average of previous scores also predicted any, violent, and sexual recidivism more strongly than baseline scores. Another recent study examined change in dynamic risk and strength scores using Service Planning Instrument (SPIn) assessments from a large community-based sample (Wanamaker & Brown, 2021). Change was examined across three to five timepoints over 30 months. Dynamic risk scores were found to gradually decrease over time and strength scores were found to gradually increase. Change in dynamic risk predicted technical violations, criminal recidivism, and violent recidivism, but change in strengths only predicted technical violations.

Emphasizing the importance of dynamic risk and strengths, a group of researchers has examined the dynamic properties in another measure, the Dynamic Risk Assessment for Offender Re-entry (DRAOR) in New Zealand (Davies et al., 2021; Lloyd et al., 2020; Muirhead, 2016; Stone et al., 2021; Stone et al., 2022). The DRAOR is a structured case management tool designed to assist probation and parole officers in the ongoing assessment of dynamic risk and strengths throughout supervision. It serves as the primary case management tool in both New Zealand and Iowa, where it was implemented in 2010 and 2014, respectively.

The research from New Zealand has demonstrated the utility of updating DROAR assessments throughout supervision, supporting its use as a dynamic risk and strength tool in this region (Davies et al., 2021; Lloyd et al., 2020; Muirhead, 2016; Stone et al., 2021; Stone et al., 2022). However, there is only one study on the dynamic properties of the DRAOR in Iowa, which did not produce favorable results (Chadwick, 2020). The purpose of the current study is to examine the validity of the DRAOR as a dynamic risk and strength tool in Iowa, United States given its widespread use across the state. To contextualize the apparent discrepancies in results, we first review the development and implementation of the DRAOR in New Zealand versus Iowa.

## DRAOR Development and Implementation

The DRAOR’s theoretical underpinnings are rooted in a model of community re-entry that presents a life-course perspective (Serin et al., 2010). The scale measures crime acquisition and desistance factors across Stable, Acute, and Protect domains. As outlined above, Stable and Acute domains reflect dynamic risk across two possible rates of change (months or years for the Stable domain vs. hours, days, or weeks for the Acute domain).

In Iowa, the DRAOR is used with individuals rated as moderate to high static risk on the Iowa Risk Assessment (Iowa Department of Corrections, 2003; see the Measures section). In New Zealand, it is used with all individuals released on parole.

## DRAOR Change Research

Lloyd et al. (2020) examined the short-term dynamic properties of the DRAOR in 3,421 adults on parole in New Zealand. Individuals in this study were followed for an average of 17.8 weeks and were reassessed on the DRAOR at one-week intervals (*N* = 68,667 assessments). Both general recidivists and nonrecidivists demonstrated positive changes on the DRAOR, with nonrecidivists showing the greatest improvements. Measurement invariance analyses indicated that these improvements were indeed related to changes in risk rather than changes in how the DRAOR was scored. Lloyd and colleagues also compared different prediction models to determine whether proximal DRAOR assessments outperformed distal assessments (i.e., those furthest in time to recidivism). Models included baseline scores, proximal scores, a simple rolling average of all scores up to each timepoint, and moving rolling averages that incorporated two to eight of the most recent scores at each timepoint. General recidivism was measured in the 1 to 2 weeks following the last assessment. Proximal DRAOR scores demonstrated incremental prediction over static risk and baseline DRAOR scores, and improved model fit compared with the average of earlier scores. Harrell’s *C*s for the best performing univariate models were small (Protect) to moderate in magnitude (Stable and Acute). The Harrell’s *C* for the multivariate model with static risk, and baseline and proximal DRAOR scores was large in magnitude.

Davies et al. (2021) replicated this study using a community-supervised sample of men rated as high risk in New Zealand (*N* = 966). The authors followed the sample for 6 months and examined weekly changes on the DRAOR. Predictive validity was tested using the same models described in Lloyd et al. (2020). Small changes in DRAOR scores were observed between baseline and final assessments for recidivists and nonrecidivists, but minimal differences were observed between groups (although, this could partly be explained by the homogeneity of the sample). Changes in DRAOR scores were related to changes in risk rather than changes in scoring, and reassessments consistently improved the prediction of imminent recidivism (over static risk and baseline DRAOR scores). Harrell’s Cs for the best performing univariate models were small in magnitude, and moderate for the multivariate model including static risk and baseline and proximal DRAOR scores.

Using the same sample as Lloyd (2020), Stone et al. (2022) investigated the predictive and incremental validity of individual trajectories of DRAOR scores. Models included (1) current score, (2) recent change score, (3) current + recent change score, and (4) overall change score. The first three models were measured across 8-week intervals (4–12 weeks, 12–20 weeks, and 24–32 weeks). The current score represents the last assessment within each interval, the recent change score represents change over each interval, and the overall change score represents the change from baseline to the current score within each interval. The study authors followed the sample for 9-week intervals (the above 8-week intervals + 1 week) to see who was reconvicted for a technical violation and/or new criminal offense. All models significantly predicted reconvictions across DRAOR domains and 8-week intervals, and AUCs were large in magnitude. The current + recent change scores also predicted reconvictions over and above each score on their own, at which point only the Stable recent change score remained statistically significant. When comparing the four DRAOR models using fit indices, the overall change score had the worst performance. Hence, clients’ current and recent presentation of risk appears more useful for predicting imminent recidivism than their entire assessment history.

Several other studies have examined the dynamic properties of the DRAOR in New Zealand with specialized populations or outcomes and have reported similar findings to the ones discussed here (Muirhead, 2016; Stone et al., 2021). However, support for the DROAR as a dynamic risk and strength tool in Iowa is lacking. In the only study of its kind in Iowa, Chadwick (2020) utilized 28,023 assessments of 3,976 adults serving community supervision orders to examine DRAOR change trajectories. On average, assessments were completed every 1 to 2 months, which resulted in 3 to 45 assessments per person. DRAOR scores changed significantly over the course of supervision, but changes were less pronounced than those observed in the New Zealand samples (change was linear for the Stable and Protect domains but quadratic for the acute domains). Higher risk individuals also demonstrated the greatest improvements on all three DRAOR domains. Measurement invariance analyses indicated that changes in DRAOR scores, however slight, were not related to changes in scoring. Chadwick also examined the predictive validity of domain change scores while controlling for baseline domain scores, age, and static risk. All change scores predicted revocations, with the overall model producing a moderate effect (Harrell’s *C* = .67). Results for criminal recidivism were less promising, as none of the change scores contributed uniquely to prediction and the overall model produced a small effect (Harrell’s *C* = .62).

## The Current Study

This research examines the dynamic properties of the DRAOR in Iowa by (1) measuring trajectories of change on the DRAOR’s three domains (Stable, Acute, Protect) over time, and (2) comparing predictive validity of baseline versus proximal DRAOR total and domain scores. For our first research question, we expect domain scores to improve over time (risks will decrease, strengths will increase) and for changes to be more pronounced for higher-risk individuals. However, we expect changes to be small given Iowa’s auto-fill system and Chadwick’s (2020) findings. Also given Chadwick’s findings, we expect changes on the Stable and Protect domain to be linear, but we make no predictions about the pattern of change for Acute scores; the Acute domain reflects destabilizers and lifestyle stressors that may fluctuate substantially from one assessment to the next (e.g., anger or being laid off), whereas the Stable and Protect domains are more enduring (e.g., attitudes, identity). For our second research question, we expect proximal DRAOR total and domain scores to predict recidivism and proximal scores will outperform baseline scores in univariate tests. However, we anticipate effect sizes will be small and smaller for criminal recidivism than for revocations, as shown in Chadwick (2020).

## Method

### Sample

The initial sample included men and women on community supervision in Iowa between October 20, 2018 and November 9, 2020 (*N* = 11,412). The sample was primarily male (82.1%) and White (74.3%), with an average age of 33 years (*SD* = 10.3) at the start of supervision. There rest of the sample identified as Black (23.1%), Indigenous (1.7%), or Asian or Pacific Islander (0.8%). For the first part of this study examining trajectories of change, as we were interested in change over time, individuals with fewer than three DRAOR assessments were excluded (*n* = 6,602). We also removed individuals under the lowest supervision level (*n* = 74, discussed in Data Cleaning section below). This brought our final sample for the first research question to 4,736 individuals with a total of 33,965 DRAOR assessments. The second research question examining predictive validity utilized the full sample (*N* = 11,412).

### Measures

#### DRAOR

The DRAOR is a 19-item case management instrument designed to aid in the ongoing assessment of dynamic risk and strengths throughout supervision (Serin, 2007). Items are scored on a 3-point scale. For Stable and Acute factors, *0 = Absence of risk factor, 1 = Slightly problematic*, and *2 = Problematic.* For Protect items, *0 = Not protective, 1 = Could be protective or not enough information*, and *2 = Protective.* Total scores are calculated in Iowa by summing the Stable and Acute domains and subtracting the Protect domain. Baseline DRAOR total and domain assessments have produced poor to fair levels of predictive validity in Iowa (*AUC*s = .53 to .62; Chadwick, 2014), and change scores have produced fair to good levels of predictive validity (Harrell’s *C*s = .62 and .67; Chadwick, 2020).

#### Time

Time reflects the number of DRAOR assessments conducted: Time 0 reflects the initial assessment, Time 1 reflects the first reassessment, and so on. Community supervision officers assessed clients on the DRAOR at the start of supervision, with follow-up assessments conducted every 3 months for the full scale, and every month for the Acute domain.

#### Supervision Level

Initial supervision level was determined using the Iowa Risk Assessment (IRA; Iowa Department of Corrections, 2003). The IRA is a 13-item primarily static risk instrument designed to aid in the prediction of general recidivism in adults on community supervision in Iowa. Total scores on the scale are used to classify individuals into one of five supervision levels (Level 1 = Administrative/Minimum, Level 2 = Low Normal, Level 3 = Normal, Level 4 = High Normal, Level 5 = Intensive). Supervision level was then adjusted based on DRAOR risk level, which is categorized as Low (≤2), Moderate (3–9), Moderate/High (10–22), or High (≥23); supervision levels were decreased by one level when DRAOR scores were Low, increased by one level when DRAOR scores were Moderate/High or High, and left the same when DRAOR scores were Moderate (Serin & Chadwick, 2017). Supervision level was also sometimes manually overridden by community supervision officers based on their professional opinion, but to facilitate analyses, we used supervision level assigned at the start of supervision that were not overridden.

#### Outcome

Community outcomes included return to custody for criminal recidivism or breach of supervision conditions (henceforth referred to as revocation; *n* = 1,570; 33.2%), or return to custody for criminal recidivism only, excluding breach of supervision conditions (*n* = 659; 13.9%). Individuals were followed from the date release until either return to custody or to the end of study for those who did not receive a new sentence or revocation (November 9, 2020). For the first research question regarding trajectories of change, a variable followup period was used. The average follow-up time was 150 days (*SD* = 124.1, *min* = 1, *max* = 657). Individuals who returned for criminal recidivism had an average time to new sentence of 152 days (*SD* = 124.0), while those revoked had an average time to revocation of 153 days (*SD* = 124.9). For the second research question regarding predictive validity, a fixed, 1 year followup was used.

### Data Analysis

#### Data Cleaning

Per Iowa policy, follow-up DRAOR assessments are optional for individuals under Level 1 and Level 2 supervision. This created an issue for analyses as our goal was to examine trajectories of change, which requires at least three DRAOR assessments per person. Presumably, officers conduct follow-up assessments with only the highest risk cases at Level 1 and Level 2 supervision, meaning low risk cases would likely have fewer than three assessments. The removal of cases with fewer than three assessments (*n* = 6,602) therefore led to the systematic removal of lower risk cases. This resulted in unusually high baseline DRAOR scores for Level 1 cases initially retained for analyses *(M* = 3.7, 3.3, 5.9, 9.9, 11.5 for Levels 1 through 5, respectively). Given that Level 1 cases with three or more assessments were not representative of all Level 1 cases in Iowa, they were removed from this study (*n* = 74).

#### Measurement Invariance

Measurement invariance was examined with a test of scalar invariance to assess whether the factor structure of the DRAOR was consistent over time (Asparouhov & Muthen, 2009). Establishing scalar invariance indicates that changes in scores over time are not attributable to changes in scoring upon reassessment (Meredith, 1993). Violation of this assumption would make interpretation of changes over time unreliable, as the change could not be uniquely attributed to changes in the construct. Scalar invariance is examined by comparing model fit between restricted models where (1) the factor loadings at each assessment are held constant and (2) where both the factor loadings and intercepts at each assessment are held constant (Putnick & Bornstein, 2016). We examined scalar invariance in DRAOR domain scores over the first three assessments for the final sample. Scalar invariance was met as there was no significant change in model fit between the restricted models.

#### Trajectories of Change

Random Intercept Longitudinal Mixed Effects models were used to examine trajectories of change in DRAOR domain scores over time, adjusting for the clustering effects of client. Mixed effects models allow for decomposition of within- and between-subjects effects: fixed effects assume no variation between individuals and random effects assume variation between groups or individuals. Model specification requires comparing model fit and visual inspection of series plots beginning with an unconditional model, an unconditional growth curve model including the fixed effect of time, and a growth curve model including the random effect of time (see Supplemental Appendices A–C for full model building).

The final models included (1) the fixed and random effects of time at Level 1, allowing for varying change over time by individual, and (2) the fixed effects of initial supervision level and the cross-level interaction between time and supervision level at Level 2. The between-subject effect of level of supervision indicates whether increase in supervision level predicts the trajectory of change in DRAOR domain scores over time. Supervision level was sequentially coded, as an ordinal predictor; the coefficient reflects change in the outcome variable from the previous level. The interaction indicates whether increase in level of supervision explains individual differences in trajectories of change in DRAOR domain scores over time.

Intraclass correlation coefficients (ICC) and design effects were calculated to investigate the necessity of a multilevel model. A significant ICC supports the use of multilevel modeling whereas an ICC of 0 indicates no variance can be attributed to between-group differences and does not. Design effects larger than 2 support the use of multilevel models (Muthén & Satorra, 1995). For this study, ICC and design effects supported the use of multilevel modeling.

There was slight non-normality in Level 1 residual domain scores for each model. A sensitivity analysis was conducted to examine the removal of potential outliers. This option was rejected, as it did not greatly change estimates or significance and would not properly reflect the observed changes. Robust standard errors were requested using the SAS EMPIRICAL command for sandwich estimators. Normality was met for Level 2 random effects. Homoscedasticity was examined with visual plots and was met in Level 1 residuals and Level 2 random effects; supporting fixed effects models. Lastly, linearity was examined for each model, reflecting our hypotheses about potential nonlinear change in Acute domain scores; results are reported below.

#### Predictive Validity

Area under the curve (*AUC*) analysis was used to examine the predictive validity of baseline and proximal DRAOR total and domain scores on the entire sample (*N* = 11,412). AUC analysis provides a measure of overall predictive accuracy of a scale instrument on a binary outcome by examining rates of true and false positives across decision thresholds (Rice & Harris, 2005). AUC values range from 0 to 1, where 1.0 is prefect predictive accuracy and 0.50 is chance predictive accuracy, wherein .56, .64, and .71 represent fair, good, and excellent levels of predictive utility, respectively.

## Results

As expected, DRAOR risk scores increased as supervision level increased, whereas DRAOR Protect scores decreased as supervision level increased (see Table 1).

**Table 1.** Mean DRAOR Subscale Scores Across Supervision Levels.

DRAOR Subscale	Level 2 *M* (*SD*)	Level 3 *M* (*SD*)	Level 4 *M* (*SD*)	Level 5 *M* (*SD*)	Overall *M* (*SD*)
Stable	5.06 (2.05)	5.78 (2.02)	7.30 (2.10)	7.72 (2.09)	6.51 (2.29)
Acute	4.79 (2.41)	5.86 (2.37)	7.41 (2.57)	8.23 (2.53)	6.61 (2.74)
Protect	6.58 (2.01)	5.79 (2.04)	4.81 (2.18)	4.47 (2.05)	5.36 (2.21)

*Note.* Stable and Protect scores range from 0 to 12 and Acute scores range from 0 to 14. Higher Acute and Stable scores indicate greater risk, whereas higher Protect scores indicate reduced risk.

### Trajectories of Change

#### DRAOR Stable Domain

Multilevel modeling was used to examine changes in DRAOR Stable scores over time by supervision level, with observations nested in clients. First, the null model was used to examine whether there was a significant effect of the clustering variable, client. The ICC indicated that approximately 83.77% of the variance was accounted for by the clustering effect of client, and the design effect was 6.17. See Supplemental Appendix A for full model building. The fixed effect of time was significant, indicating that clients’ Stable scores increased by an average of 0.06 points with every reassessment, controlling for the other factors in the model (see Table 2). It is important to note there was an observed suppression effect in model building. When the interaction between reassessment and supervision was included in the model, the coefficient for time changed direction from −0.06 to 0.06. Thus, the expected direction of this coefficient was observed, but changed with the full model.

**Table 2.** Model for Criterion DRAOR Stable Subscale Scores.

	*Est*.	*SE*	*p*	CI [95%]	
Model				LL	UL
Fixed effects					
Intercept (γ_00_)	4.57	0.09	<.001	4.39	4.75
Time (γ_10_)	0.06	0.02	.002	0.02	0.09
Level 3 (γ_01_)	0.93	0.81	<.001	0.77	1.09
Level 4 (γ_02_)	1.30	0.05	<.001	1.19	1.41
Level 5 (γ_03_)	0.98	0.06	<.001	0.86	1.10
Time × Level 3 (γ_11_)	−0.05	0.01	.001	−0.08	−0.02
Time × Level 4 (γ_12_)	−0.07	0.01	<.001	−0.09	−0.04
Time × Level 5 (γ_13_)	−0.004	0.01	.747	−0.03	0.02
Random effects					
Intercept ( $\tau_{0}^{2}$ )	4.44	0.09	<.001	4.23	4.63
Residual (σ^2^)	0.37	0.003	<.001	0.36	0.38
Slope of Time ( $\tau_{1}^{2}$ )	0.07	0.002	<.001	0.06	0.07

*Note.* The criterion reflects DRAOR Stable subscale scores. Stable subscale scores range from 0 to 12 (*Low = 0–1, Moderate = 2–6, High = 7–12*), wherein greater scores indicate greater risk. Time reflects the Time an individual was assessed by the DRAOR, beginning with their initial assessment on supervision (*Time = 0*), followed by up to 11 reassessments. Supervision level was sequential coded.

The fixed effects of supervision levels were also significant. Recall the predictors were sequentially coded and thus interpretation compares changes from the previous supervision level. Clients supervised at Level 3 had Stable scores 0.93 points higher, on average, than individuals supervised at Level 2, controlling for the other factors in the model. Clients supervised at Level 4 had Stable scores 1.30 points higher, on average, than individuals supervised at Level 3, controlling for the other factors in the model. Clients supervised at Level 5 had Stable scores 0.98 points higher, on average, than individuals supervised at Level 4, controlling for the other factors in the model. Lastly, the interactions between time and Levels 3 and 4 supervision were significant while controlling for the other factors in the model. Stable scores for individuals supervised at Level 3 decreased by 0.05 points more with each reassessment than individuals supervised at Level 2. Similarly, Stable scores for individuals supervised at Level 4 decreased by 0.07 points more with each assessment for those supervised at Level 3.

#### DRAOR Acute Domain

Following the same steps as above, multilevel modeling was supported: the ICC indicated that approximately 59.71% of the variance was accounted for by the clustering effect of client, and the design effect was 4.68. See Supplemental Appendix B for full model building. The fixed effect of time was significant, indicating that clients’ Acute scores increased by an average of 0.32 points with every reassessment, controlling for the other factors in the model (see Table 3). Of note, the pattern of change observed for this effect was quadratic. Like the Stable model, there was a suppression effect for this coefficient with the added interaction term between reassessment and supervision (i.e., −0.32 changed to 0.32).

**Table 3.** Model for Criterion DRAOR Acute Subscale Scores.

	*Est*.	*SE*	*p*	CI [95%]	
Model				LL	UL
Fixed effects					
Intercept (γ_00_)	3.97	0.13	<.001	3.72	4.22
Time (γ_10_)	0.32	0.03	.235	−0.02	0.09
Time × Time (γ_20_)	0.01	0.001	<.001	0.003	0.01
Level 3 (γ_01_)	1.34	0.11	<.001	1.12	1.55
Level 4 (γ_02_)	1.84	0.06	<.001	1.72	1.96
Level 5 (γ_03_)	1.65	0.07	<.001	1.50	1.79
Time × Level 3 (γ_11_)	−0.06	0.02	.003	−0.10	−0.02
Time × Level 4 (γ_12_)	−0.13	0.01	<.001	−0.16	−0.11
Time × Level 5 (γ_13_)	−0.03	0.01	.038	−0.06	−0.002
Random effects					
Intercept ( $\tau_{0}^{2}$ )	5.98	0.13	<.001	5.73	6.24
Residual (σ^2^)	0.87	0.01	<.001	0.85	0.89
Slope of Time ( $\tau_{1}^{2}$ )	0.12	0.004	<.001	0.12	0.13

*Note.* The criterion reflects DRAOR Acute subscale scores. Acute subscale scores range from 0 to 12 (*Low = 0–1, Moderate = 2–7, High = 8–14*), wherein greater scores indicate greater risk. Time reflects the Time an individual was assessed by the DRAOR, beginning with their initial assessment on supervision (*Time = 0*), followed by 11 reassessments. Interactions were conducted with linear time for interpretability. Supervision level was sequential coded.

The fixed effects of supervision level were also significant. Clients supervised at Level 3 had Acute scores 1.34 points higher, on average, than individuals supervised at Level 2, controlling for the other factors in the model. Clients supervised at Level 4 had Acute scores 1.84 points higher, on average, than individuals supervised at Level 3, controlling for the other factors in the model. Clients supervised at Level 5 had Acute scores 1.65 points higher, on average, than individuals supervised at Level 4, controlling for the other factors in the model. Lastly, the interaction terms between time and Levels 3 and 4 supervision were significant while controlling for the other factors in the model. These interactions were conducted with linear time for interpretability, despite that quadratic time was a better fit for this predictor. Acute scores for individuals supervised at Level 3 decreased by 0.06 points more with each reassessment than individuals supervised at Level 2, and Acute scores for individuals supervised at Level 4 decreased by 0.13 points more with each assessment for those supervised at Level 3. The interaction between time and Level 5 was nonsignificant.

#### DRAOR Protect Domain

Following the same steps as above, multilevel modeling was supported: the ICC indicated that approximately 83.91% of the variance was accounted for by the clustering effect of client, and the design effect was 6.18. See Supplemental Appendix C for full model building. The fixed effect of time was significant, indicating that clients’ Protect domain scores decreased, on average, by 0.07 points with every reassessment, controlling for the other factors in the model (see Table 4). Like the Stable and Acute models, there was a suppression effect for this coefficient with the added interaction term between reassessment and supervision (i.e., 0.04 changed to −0.07).

**Table 4.** Model for Criterion DRAOR Protect Subscale Scores.

	*Est*.	*SE*	*p*	CI [95%]	
Model				LL	UL
Fixed Effects					
Intercept (γ_00_)	6.94	0.10	<.001	6.74	7.14
Time (γ_10_)	−0.07	0.02	<.001	−0.10	−0.03
Level 3 (γ_01_)	−0.78	0.09	<.001	−0.96	−0.61
Level 4 (γ_02_)	−1.08	0.06	<.001	−1.20	−0.97
Level 5 (γ_03_)	−0.85	0.07	<.001	−0.97	−0.72
Time × Level 3 (γ_11_)	0.06	0.02	<.001	0.03	0.09
Time × Level 4 (γ_12_)	0.06	0.01	<.001	0.04	0.08
Time × Level 5 (γ_13_)	−0.00	0.01	.809	−0.03	0.02
Random effects					
Intercept ( $\tau_{0}^{2}$ )	4.58	0.10	<.001	4.40	4.78
Residual (σ^2^)	0.36	0.003	<.001	0.35	0.37
Slope of Time ( $\tau_{1}^{2}$ )	0.07	0.002	<.001	0.06	0.07

*Note.* The criterion reflects DRAOR Protect subscale scores. Protect subscale scores range from 0 to 12 (*Low = 0–4, Moderate = 5–7, High = 8–12*), wherein greater scores indicate reduced risk. Time reflects the Time an individual was assessed by the DRAOR, beginning with their initial assessment on supervision (*Time = 0*), followed by 11 reassessments. Supervision level was sequential coded.

The fixed effects of supervision level were also significant. Clients supervised at Level 3 had Protect scores 0.78 points lower, on average, than individuals supervised at Level 2, controlling for the other factors in the model. Clients supervised at Level 4 had Protect scores 1.08 points lower, on average, than individuals supervised at Level 3, controlling for the other factors in the model. Clients supervised at Level 5 had Protect scores 0.85 points lower, on average, than individuals supervised at Level 4, controlling for the other factors in the model. Lastly, the interaction terms between time and Levels 3 and 4 were significant while controlling for the other factors in the model. Protect scores increased by 0.06 points more with each assessment for those supervised at Level 3 versus Level 2 and Level 4 versus Level 3. The interaction between time and Level 5 was nonsignificant.

### Predictive Validity of Baseline Versus Proximal DRAOR Scores

Predictive validity results for DRAOR baseline versus proximal scores are displayed in Table 5. In predicting criminal recidivism, DRAOR baseline total and domain scores produced fair levels of predictive validity (*AUC* = .58–.61), but proximal scores were not predictive, likely due to small baserates (*n* = 28). In predicting revocation, DRAOR baseline total and domain scores produced fair to good levels of predictive validity (*AUC* = .63–.67) and proximal scores produced good levels (*AUC* = .64–.69). For revocation, the confidence intervals overlapped, indicating that the improvement in proximal scores was not significantly better than baseline scores (Cumming & Finch, 2005).

**Table 5.** Predictive Validity of DRAOR Scores on Time to Criminal Recidivism and Revocation.

	*N*	Baseline			Proximal		
Model		*n*	*AUC*	*CI* 95% [*LL, UL*]	*n*	*AUC*	*CI* 95% [*LL, UL*]
Time to criminal recidivism	2,275	503			28		
Stable			.58	[.57, .59]		NS	
Acute			.59	[.57, .60]		NS	
Protect			.61	[.60, .63]		NS	
DRAOR total			.61	[.60, .63]		NS	
Time to revocation	2,275	411			237		
Stable			.63	[.61, .65]		.64	[.61, .67]
Acute			.65	[.63, .67]		.67	[.64, .70]
Protect			.63	[.62, .65]		.67	[.63, .70]
DRAOR total			.67	[.65, .68]		.69	[.65, .72]

*Note.* NS = non significant.

## Discussion

In the current study, we examined the dynamic properties of the DRAOR in Iowa. Our main research question asked if DRAOR domain scores change over time, and whether this change differs by risk level (measured as supervision level). We found significant improvement in DRAOR domain scores over time, as was seen in a prior study in Iowa (Chadwick, 2020); this was accords with observations in New Zealand (Lloyd et al., 2020), but with smaller magnitude.

As predicted, improvements in domain scores over time were more pronounced for individuals classified into higher versus lower supervision levels. This makes sense from both a supervision and a statistical perspective. We would expect individuals at higher risk levels to receive more intensive interventions, improving their domain scores over time. We would also expect that individuals at higher risk levels have more change to be observed in a single direction, as they already sit at the rating ceiling. Recall DRAOR items are rated on a 3-point scale, limiting sensitivity in observations of change. An individual who sits in the middle of the risk relevant scale (*0 = Absence of risk factor, 1 = Slightly problematic*, and *2 = Problematic*) would need to reflect an absence of that factor, rather than improvement to reflect change.

Baseline and proximal DRAOR total and domain scores were good predictors of time to revocation in this sample. Similar to the New Zealand sample (Lloyd et al., 2020) proximal assessments were better predictors of criminal recidivism than baseline, but only slightly. While previous research in New Zealand supports use of proximal assessments to best predict recidivism (Lloyd et al., 2020, Stone et al., 2022), the lack of overall change in this sample suggests the differences were not substantial in Iowa.

Practically speaking, the findings in this study can inform supervision practice. The DRAOR as a case management tool does predict criminal recidivism and revocation in this sample. Additionally, it also supports stronger intervention at higher risk level, though the effects were small. The lack of change in scores over reassessment suggest there may be a lack of fidelity to reassessment. True reassessment is expected to garner a more accurate picture of current risk to inform intervention strategy. Conclusions regarding this point are made below.

## Conclusions

While clients demonstrated statistically significant changes on the DRAOR domains over time, changes were small in magnitude. As criminal recidivism and revocation base rates did not increase over time, this may be an artifact of implementation fidelity. Despite significant enhancements in training, two issues may have impacted change scores. First, Iowa’s autofill scoring system for the DRAOR is reducing the amount of change officers record from one assessment to the next, except in exceptional circumstances. Second, recent quality assurance reviews indicated that a not insignificant number of staff failed to complete reassessments according to policy (S. Kreamer, personal communication, 26 April 2023) meaning the autofill became the default. Iowa’s training practices are likely another contributing factor to the poor implementation fidelity of the DRAOR. Since the time of this data collection, this jurisdiction has improved its training and procedures. Namely, training has evolved to increase reliability with a robust e-learning curriculum and increased oversight (S. Kreamer, personal communication, August 17, 2022).

There are several other important limitations to this research. First, we excluded clients supervised at Level 1 because, after removing those with fewer than three assessments, the group was not representative of all Level 1 cases in Iowa. This precluded comparisons between clients supervised at Level 1 versus 2 and may have also influenced the overall results. We also elected to examine the calculated supervision level for these analyses and not the supervision levels after manual overrides, given concerns about overrides (Cohen et al., 2020). While only 183 assessments in the final sample had a supervision level override, this decision may reduce validity as individuals may have been supervised at a higher or lower level than was recorded here. More importantly, by reducing the sample to include only those with reassessments, we sacrificed inclusion of individuals who may have failed sooner. Research suggests such individuals would be in the higher risk category (Lowenkamp et al., 2016). As a result, these findings should be viewed in the context of excluding both low risk, with exclusion of Level 1 supervision, and high risk reoffenders, leaving mostly moderate risk individuals, or individuals at higher risk who did not reoffend or reoffended later. A strength of this study was the statistical approach. Literature examining risk assessment using dynamic risk items should examine change with proper accounting for measurement invariance and potential clustering effects within individuals with repeated measures.

## Supplemental Material

sj-docx-1-ijo-10.1177_0306624X241240701 – Supplemental material for Examining Trajectories of Change on the Dynamic Risk Assessment for Offender Re-Entry (DRAOR) Supplemental material, sj-docx-1-ijo-10.1177_0306624X241240701 for Examining Trajectories of Change on the Dynamic Risk Assessment for Offender Re-Entry (DRAOR) by Danielle J. Rieger, Bronwen Perley-Robertson and Ralph C. Serin in International Journal of Offender Therapy and Comparative Criminology
