# Supplementary material for: Examining Trajectories of Change on the Dynamic Risk Assessment for Offender Re-Entry (DRAOR)
Source: Int J Offender Ther Comp Criminol. 2024 Apr 5;70(6-7):630–47. doi: 10.1177/0306624X241240701 (PMC13002922; doi:10.1177/0306624X241240701)
Supplement: sj-docx-1-ijo-10.1177_0306624X241240701 – Supplemental material for Examining Trajectories of Change on the Dynamic Risk Assessment for Offender Re-Entry (DRAOR) [file sj-docx-1-ijo-10.1177_0306624X241240701.docx]

**Appendix A**

*Model Building for Criterion DRAOR Stable Subscale Scores*

|  | Model 0 | |  | Model 1 | |  | Model 2 | | |  |
| --- | --- | --- | --- | --- | --- | --- | --- | --- | --- | --- |
|  | Est.(*SE*) | CI [95%] |  | Est.(*SE*) | CI [95%] |  | Est.(*SE*) | CI [95%] | |  |
| Fixed Effects |  |  |  |  |  |  |  |  | |  |
| Intercept | 6.28(0.03)* | [6.23, 6.34] |  | 6.40(0.03)* | [6.35, 6.46] |  | 6.40(0.03)* | | [6.35, 6.46] | |
| Time |  |  |  | -0.05(0.01)* | [-0.06, -0.05] |  | -0.05(0.01)* | | [-0.06, -0.04] | |
| Random Effects |  |  |  |  |  |  |  |  | |  |
| Intercept (*τ*_00_) | 5.21(0.10)* | [5.03, 5.40] |  | 5.29(0.10)* | [5.10, 5.48] |  | 6.00(0.11)* | [5.79, 6.22] | |  |
| Residual (σ^2^) | 0.90(0.01)* | [0.88, 0.91] |  | 0.87(0.01)* | [0.86, 0.89] |  | 0.44(0.00)* | [0.44, 0.45] | |  |
| Covariance (*τ*_01_) |  |  |  |  |  |  | -0.23(0.01)* | [-0.26, -0.21] | |  |
| Slope of Time (*τ*_11_) |  |  |  |  |  |  | 0.09(0.00)* | [0.09, 0.10] | |  |
| Deviance | 122,880.2 |  |  | 122,124.9 |  |  | 110,772.0 |  | |  |
| Decrease in deviance |  |  |  | 755.3* |  |  | 11352.9* |  | |  |

*Note*. Model 0 = Null model. Model 1 = Unconditional growth curve model. Model 2 = Unconditional growth curve model with random intercept of Time.

** p* < .001.

**Appendix B**

*Model Building for Criterion DRAOR Acute Subscale Scores*

|  | Model 0 | |  | Model 1 | |  | Model 2 | | |  |
| --- | --- | --- | --- | --- | --- | --- | --- | --- | --- | --- |
|  | Est.(*SE*) | CI [95%] |  | Est.(*SE*) | CI [95%] |  | Est.(*SE*) | CI [95%] | |  |
| Fixed Effects |  |  |  |  |  |  |  |  | |  |
| Intercept | 6.42(0.03)* | [6.36, 6.48] |  | 6.64(0.03)* | [6.57, 6.71] |  | 6.65(0.03)* | | [6.58, 6.72] | |
| Time |  |  |  | -0.09(0.01)* | [-0.11, -0.08] |  | -0.10(0.01)* | | [-0.11, -0.09] | |
| Random Effects |  |  |  |  |  |  |  |  | |  |
| Intercept (*τ*_00_) | 6.22(0.12)* | [6.00, 6.46] |  | 6.36(0.12)* | [6.13, 6.61] |  | 7.32(0.14)* | [7.06, 7.59] | |  |
| Residual (σ^2^) | 1.98(0.02)* | [1.94, 2.00] |  | 1.89(0.02)* | [1.86, 1.92] |  | 1.04(0.01)* | [1.02, 1.06] | |  |
| Covariance (*τ*_01_) |  |  |  |  |  |  | -0.35(0.02)* | [-0.39, -0.31] | |  |
| Slope of Time (*τ*_11_) |  |  |  |  |  |  | 0.17(0.01)* | [0.16, 0.18] | |  |
| Deviance | 148,133.0 |  |  | 147051.9 |  |  | 137,024.1 |  | |  |
| Decrease in deviance |  |  |  | 1081.1* |  |  | 10027.8* |  | |  |

*Note*. Model 0 = Null model. Model 1 = Unconditional growth curve model. Model 2 = Unconditional growth curve model with random intercept of Time.

** p* < .001.

**Appendix C**

*Model Building for Criterion DRAOR Protect Subscale Scores*

|  | Model 0 | |  | Model 1 | |  | Model 2 | | |  |
| --- | --- | --- | --- | --- | --- | --- | --- | --- | --- | --- |
|  | Est.(*SE*) | CI [95%] |  | Est.(*SE*) | CI [95%] |  | Est.(*SE*) | CI [95%] | |  |
| Fixed Effects |  |  |  |  |  |  |  |  | |  |
| Intercept | 5.46(0.03)* | [5.41, 5.51] |  | 5.35(0.03)* | [5.29, 5.40] |  | 5.35(0.03)* | | [5.30, 5.41] | |
| Time |  |  |  | 0.05(0.00)* | [0.04, 0.06] |  | 0.04(0.01)* | | [0.03, 0.05] | |
| Random Effects |  |  |  |  |  |  |  |  | |  |
| Intercept (*τ*_00_) | 4.72(0.09)* | [4.56, 4.90] |  | 4.75(0.09)* | [4.59, 4.94] |  | 5.41(0.10)* | [5.23, 5.61] | |  |
| Residual (σ^2^) | 0.84(0.01)* | [0.82, 0.85] |  | 0.82(0.01)* | [0.80, 0.83] |  | 0.43(0.01)* | [0.42, 0.43] | |  |
| Covariance (*τ*_01_) |  |  |  |  |  |  | -0.22(0.01)* | [-0.24, -0.20] | |  |
| Slope of Time (*τ*_11_) |  |  |  |  |  |  | 0.09(0.00)* | [0.08, 0.09] | |  |
| Deviance | 120059.2 |  |  | 119398.0 |  |  | 108778.3 |  | |  |
| Decrease in deviance |  |  |  | 661.2* |  |  | 10619.7* |  | |  |

*Note*. Model 0 = Null model. Model 1 = Unconditional growth curve model. Model 2 = Unconditional growth curve model with random intercept of Time.

** p* < .001.
